# Using an innovative stacked ensemble algorithm for the accurate prediction of preterm birth

**DOI:** 10.4274/jtgga.galenos.2018.2018.0105

**Published:** 2019-05-28

**Authors:** Pari Ramalingam, Maheshwari Sandhya, Sharmila Sankar

**Affiliations:** 1 Department of Computer Science and Engineering, B. S. Abdur Rahman Crescent Institute of Science and Technology, Chennai, India

**Keywords:** Preterm birth, neonatal death, risk factors of preterm birth, stacked ensemble, stacked generalization, meta-learning

## Abstract

**Objective:**

A birth before the normal term of 38 weeks of gestation is called a preterm birth (PTB). It is one of the major reasons for neonatal death. The objective of this article was to predict PTB well in advance so that it was converted to a term birth.

**Material and Methods:**

This study uses the historical data of expectant mothers and an innovative stacked ensemble (SE) algorithm to predict PTB. The proposed algorithm stacks classifiers in multiple tiers. The accuracy of the classiffication is improved in every tier.

**Results:**

The experimental results from this study show that PTB can be predicted with more than 96% accuracy using innovative SE learning.

**Conclusion:**

The proposed approach helps physicians in Gynecology and Obstetrics departments to decide whether the expectant mother needs treatment. Treatment can be given to delay the birth only in patients for whom PTB is predicted, or in many cases to convert the PTB to a normal birth. This, in turn, can reduce the mortality of babies due to PTB.

## Introduction

Births that happen after 37 weeks of gestation and before 39 weeks are termed as normal birth (TB). Babies born before 37 weeks of gestation are considered as premature babies and such births are termed as preterm birth (PTB) (1,2). Premature babies typically have many severe complications such as breathing/respiratory problems (apnea), chronic lung disease, jaundice, anemia, infections, bleeding in the brain (intraventricular hemorrhage). In the worst cases, premature babies die in the early days of life. Such deaths are termed as neonatal death (3). The United Nations International Children's Emergency Fund report published in 2015 stated that PTB was a major cause of the neonatal death (4,5,6). Due to PTB, some women also have poor mental health, and in some extreme cases have mental disorders (7). The long-term consequences of PTB for the babies are cognitive problems (intellectual disability and learning disability), asthma, intestinal problems, vision problems, hearing loss problems, dental problems, poor growth, and increased risk of sudden infant death syndrome.

When the expectant mother undergoes prenatal checkups, the clinical pathologic status may indicate the possibility of a PTB. In the Obstetrics and Gynecology (O&G) world, these indicators are called risk factors of PTB (8,9,10,11). The physician analyzes these risk factors and diagnoses the birth as either TB or PTB. While diagnosing PTB, the physician also takes into consideration the behavioral and social characteristics of the expectant mother (12). Hence, they are also considered as risk factors of PTB. All risk factors are not critical in nature and they do not contribute equally to PTB. Hence, risk factors are categorized as primary risk factors and secondary risk factors based on their criticality. The primary and secondary risk factors associated with PTB are listed in Table 1.

Obtaining evidence for PTB in clinical pathology is a challenging task. More than that, some clinical tests are too expensive to for patients from developing countries. Accordingly, predictive analytics is the way forward. Predicting a PTB as a TB can lead to fatal consequences, thus learning algorithms with high accuracy are very much needed. Ensemble learning gives better accuracy than individual learning algorithms and hence is suitable for predicting PTB (15,16). Ensembles perform effectively, especially if the base learners are diverse and are moderately performing (17). Using a trainable combiner to learn from the predictions of base learners generalizes better than traditional ensembles (18). Such learning systems are termed as stacked ensemble (SE) systems (19,20). They use base classifiers to train level-0 models and a generalizer to learn from the predictions of level-0 models. Thus, the predictions of base classifiers form the input space for the generalizer. These predictions are termed as meta-features and the generalizer is said to perform meta-learning (21,22,23).

This study uses an innovative SE algorithm for the accurate prediction of PTB. It differs from traditional SEs in producing the meta-features. Rather than using the predictions of level-0 models as meta-features, it combines them using multiple combination schemes to produce meta-features. The meta-features along with the critical features are used to train the generalizer. The combination schemes produce the joint distributions of the level-0 predictions. The predictions from level-0 models are the abstraction of the mapping between the input space and the actual labels. Hence, these joint distributions map the level-0 predictions to the actual label and indirectly map the input space with the actual label. This in turn produces meta-features that better abstracts the relationship between the input space and the actual labels. In doing so, the proposed algorithm performs better than traditional SE algorithms. The performance of the algorithm is measured using its accuracy and recall.

The following are the contributions of this study: (i) the introduction of an innovative SE algorithm to improve prediction accuracy, (ii) the algorithm enables accurate predictions of PTB, and (iii) motivation for the research community to use this algorithm for classification problems. Organization of the remaining sections: Section II describes the work conducted in predicting PTB. Section III depicts the proposed algorithm in detail. Experimental results along with the inferences are reported in section IV. Section V is the conclusion and the scope for future work.

### Related work

Using machine learning or statistical analysis for predicting PTB based on historical data is gaining momentum in O&G. Bittar et al. (24) used statistical analysis to predict spontaneous PTB for a high-risk group of expectant mothers who had prior PTB. They used the cervical length and the level of protein-1 (phIGFBP-1) in cervical secretions as the input for their statistical analysis. In the first step, they used phIGFBP-1 and cervical length to perform the logistic regression analysis to predict PTB. For examining the contributions of these two factors on PTB, they performed multiple logistic regression analysis. A dataset with 105 expectant singleton mothers were used for their analysis. They found that phIGFBP-1 level measured during the 30^th^ week of gestation helped in predicting PTB accurately. In combination with this, measuring the cervical length between the 22^nd^ and 24^th^ weeks and using it for the statistical analysis improved the prediction rate of PTB. They achieved a prediction rate of 92% before the 34^th^ week and a prediction rate of 80% before the 37^th^ week. Though their study resulted in high prediction rate, the number of PTB instances in the dataset was only 12. Hence generalizing their result involves an element of risk.

A similar kind of study was conducted by Care et al. (25) to predict PTB in women with prior PTB and normal cervical length (>25 mm) between 22-24 weeks. They used a dataset with 196 instances out of which 134 patients had a normal cervical length and 62 patients had shorter cervical length. Out of the 134 patients with normal cervical length, 28 patients had a PTB and 12 of these had a prior PTB. Of the 62 patients with shorter cervical length, 25 patients had a PTB. All these patients were from the White British population demographic. They used SPSS to conduct analyses, which revealed that a normal cervix or long cervix did not provide any assurance on recurrence of PTB. They concluded that that normal cervical length and the demographic information of the patients were not good features to predict PTB. They also suggested using other factors such as amniotic fluid, vaginal discharge, genetic, and social and environmental factors to predict PTB.

Predicting PTB outside of the clinical pathology is a better approach for the early prediction of PTB. Catley et al. (26) used back propagation feed forward Artificial Neural Network (ANN) for predicting PTB. They used Perinatal Partnership Program of Eastern and Southeastern Ontario databases for conducting the experiments. The dataset was skewed towards TB and hence they removed the TB instances to balance the dataset. While dividing the dataset into a training set and a test set, they ensured that the distribution of TB and PTB was not skewed. They used a logarithmic sensitivity index for measuring the performance of the prediction. They used MatLab with the Neural Network Toolkit to conduct the experiment. The authors used the weight-elimination cost function to improve the classification performance, and one hidden layer with three hidden nodes. When the skewed dataset was used, the sensitivity reached a peak at 20.4%. The sensitivity of the prediction reached a peak at 33.4% with a more balanced dataset. They observed that the number of fetuses in the womb in the current pregnancy and previous pregnancies, number of children, and smoking after 20 weeks of gestation were the factors with the highest connection weights in the ANN. Hence, these were major contributors for PTB.

Analyzing electrohysterography (EHG) signals for predicting PTB is another popular approach. This approach uses the signals generated by the contractions and expansions of the uterus. Ren et al. (27) used EHG signals to classify births as TB or PTB. They used the EHG signals of 300 patients available in the PhysioBank. Though the signal had a wide frequency spectrum, they filtered the frequency range of 0.3 Hz to 3 Hz. The signal was decomposed into intrinsic mode functions using empirical mode decomposition. The first ten functions were selected for prediction. They used the Gabriel Rilling EMD toolbox for this purpose. The dataset had 262 patients with TB and 38 patients with PTB. The dataset was balanced using SMOTE. They used principal component analysis to select the components such that it could improve area under the curve (AUC) values. They used multiple classifiers to classify PTB. On average, they achieved a maximum AUC value of 86.2%. They analyzed the impact of using the features from 3 channels of EHG against using only the features from channel 3. When the features from only channel 3 were used, the AUC reached a maximum of 89%. Among the classifiers, AdaBoost reached the highest accuracy of 98.6%. The limitation of this approach is the availability of EHG signals of patients in developing countries.

There are three types of features that can be extracted from uterine EHG signals. They are linear analysis; non-linear analysis and Discrete Cosine Transform analysis. Naeem et al. (28) extracted three types of features and analyzed them using ANN. They used three different forms of ANNs such as cascade-forward back propagation network, feed-forward network, and Kohonen network. The authors trained the networks with linear features, non-linear features, DCT features, and a combination of these features. They used PCA to select 10 components and used them for prediction. They compared the results obtained for all the types of features with three different forms of ANNs and concluded that the accuracy reached a peak value of 90% when feed-forward network with the linear and DCT features of uterine EHG signals were used. After analyzing the consequences of false positives, the accuracy of 90% is not enough for PTB.

The use of SE has found mention in many studies. Wang et al. (29) applied SE on a dataset constructed by Chou and Elrod (30) to predict membrane protein types based on pseudo-amino acid composition. Support Vector Machine (SVM) and Instance-Based Learning were used as level-0 classifiers. A combination of these two classifiers provided more information about the input space and its relationship with the class label. To achieve faster training, a Sequential Minimal Optimization (SMO) algorithm was used to train SVM. These classifiers were cross-validated using k-fold cross-validation. The cross-validated predictions from level-0 SVMs were used as input level-1 generalization. A Decision Tree was used as level-1 generalizer. Re-substitution test, jack-knife test and the independent dataset test were used to examine the quality of prediction through bias and variance estimation. Among the three tests, a high success rate of 85.4% was achieved in the jack-knife test. SE has achieved remarkably good performance and thus helped in providing the direction for functionally characterizing gene products using gene sequences.

## Material and Methods

When the traditional SE is used, the level-1 generalizer inherits some level of bias and variance from level-0 models. Hence the problem of overfitting or underfitting is not eliminated to the maximum possible extent. To address this issue, this study proposes an innovative SE algorithm by stacking classifiers in multiple tiers (31). The proposed algorithm stacks the classifiers in three tiers namely (i) base tier, (ii) ensemble tier, and (iii) generalization tier (32). The base tier focuses on training a set of suitable learners to achieve moderate accuracy. The second tier uses a set of combinations schemes to combine the predictions from the base learners. The outputs from the combination schemes form the input space for the next tier. The third tier does the meta-learning using the newly formed input space. The performance of this algorithm is optimized using a suitable number of base learners and a suitable number of combination schemes. The choice of meta-learner in the third tier also plays a vital role in improving the accuracy of this algorithm. The base tier ensures a reduction of bias, the ensemble tier and the generalization tier ensure the reduction of variance. As a result, the bias is perfectly balanced with the variance. Due to this, the proposed algorithm improves the classification accuracy. Hence this algorithm is suitable for classifying PTB based on the historical data of expectant mothers.

**Figure 1:**
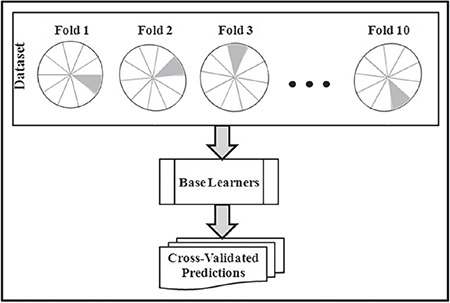
Training the base learners – base tier

In general, for any classifier, cross-validation helps in reducing bias (33,34). In the proposed algorithm, a 10-fold cross validation is also used to train the base learners. The dataset is partitioned into 10 disjoint sets. Each of these 10 sets is used one after the other as a test set. Each fold is used nine times as a training set. As a result, the base learners produce the cross-validated predictions. Figure 1 depicts the cross-validation of the base learners. The output of the base tier is multiple sets of cross-validated predictions because this process is repeated for each base learner. This serves as the input for the next tier.

**Figure 2:**
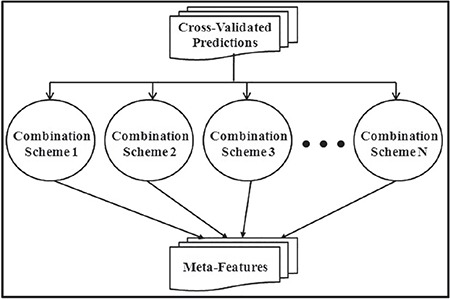
Combining the predictions from the base learners – ensemble tier

As depicted in Figure 2, the set of cross-validated predictions from each base learner are used as input in the second tier. The goal of the second tier is to combine the predictions from the base tier and to map them with one of the class labels. Hence, it creates a joint distribution of the base learners’ predictions. The output from each combination scheme provides a meta-feature. The quality of the meta-features depends on the choice of the combination schemes used in this tier. The better the combination schemes, the better the meta-features capture the inherent relationship between the input space and the actual labels. Therefore, the meta-features play an important role in the accuracy of this innovative SE algorithm. The combination schemes to be used in this tier are decided depending upon the problem on hand. Popular combination schemes such as averaging and majority voting work well for most of problems.

**Figure 3:**
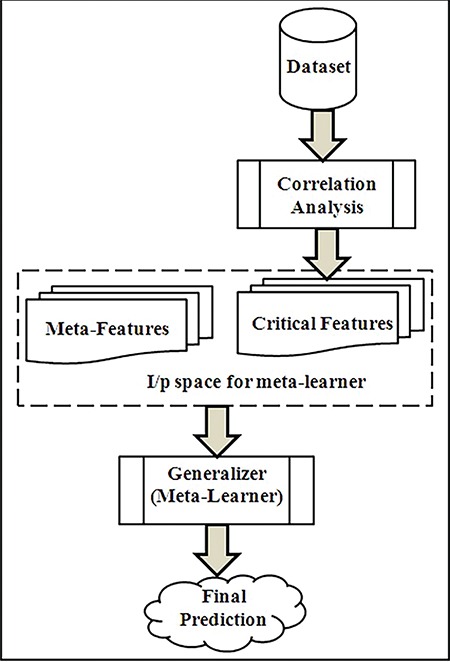
Training the meta-learner – generalization tier

As depicted in Figure 3, the meta-features and the top three critical features selected from the original input space form the input space for the meta-learner. The top three features are selected by analyzing the correlation of each feature with the class labels. The features that have high correlation with the class label are selected.

After analyzing the historical data of the patients, we decided to use the following list of base learners, the combination schemes, and the meta-learner in this study. This is shown in Table 2.

The experiment was implemented using the Python and Scikit-learn library (35). A dataset consisting of the historical data of 2600 patients was used to carry out this study. The dataset was a masked dataset without any reference to the personal details of the patients. Accordingly, the need to obtain informed consent and ethics committee approval did not arise. The details about the data set are given in Table 3. The data were thoroughly reviewed to check if the dataset had a good mix of all the possible cases: (i) mother with risk factors and had a PTB, (ii) mother without risk factors and had a PTB, (iii) mother with risk factors but had a TB, and (iv) mother without risk factors and had a TB. This mix of all the possible cases was also ensured in the training and testing data.

**Figure 4:**
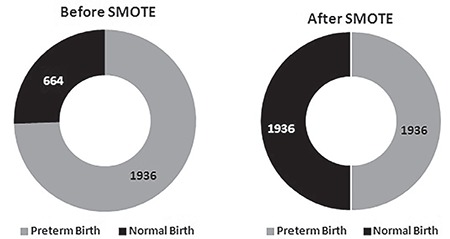
Count of NB and preterm birth in the dataset

The distribution analysis of the class labels in the dataset reveals that the dataset was asymmetric one and skewed towards TB. PTB was the minority class and TB was the majority class. Hence, the SMOTE (Synthetic Minority Over-sampling Technique) algorithm was used to balance the dataset. Balancing the dataset increases the number of minor instances to match with the number of major instances. This in turn increases the total number of instances. The count of TB and PTB in the dataset before and after SMOTE is shown in Figure 4.

The dataset was thoroughly analyzed for missing data or null values because missing data plays a large role in pulling down the accuracy of models. If missing data or null values were found in any of the features, the criticality of the feature in which it is found was analyzed. For all critical features, mean values were used to replace missing data. For all non-critical features, the default values were used. A scatter plot of the historical data was plotted to reveal the outliers. The top 5 critical features were selected and concatenated because most of the features were binary in nature. The concatenated feature is taken along the x-axis and the class label is taken along the y-axis. The central mass of the plot was identified and the points that were further away from this central mass were analyzed to identify the outliers. The identified outliers were removed from the dataset. Normalizing the dataset also helps in improving the performance of the learning algorithm. Accordingly, different normalization methods were analyzed to select a suitable one for the problem on hand. In this study, the dataset was normalized by performing mean cancellation.

To assess the impact of primary and secondary factors on the classification accuracy, multiple experiments were conducted with different subsets of features in the dataset. The list of experiments conducted is depicted in Table 4. These experiments help to understand the contributions of primary and secondary risk factors for PTB. Each experiment was repeated 10 times to ensure the consistency of the results. The average values of the accuracy, precision, recall, and the F-1 score across the trials are reported in this study. Receiver operating characteristic (ROC) curves were also drawn for each experiment and these are also reported in this study.

## Results

A comparative analysis of different performance metrics for SE and the proposed algorithm was conducted. In addition, analysis of how the feature subsets improved or degraded the performance metrics was also performed. This analysis helps in understanding the factors that make a major contribution to PTB. The results reveal that the performance metrics reached the maximum when all the features in the dataset were used for training the algorithms. Irrespective of the number of risk factors used for training, the performance of the proposed algorithm is better than the performance of SE. The results of the experiments in which all the risk factors are used for training is summarized in Table 5.

**Figure 5:**
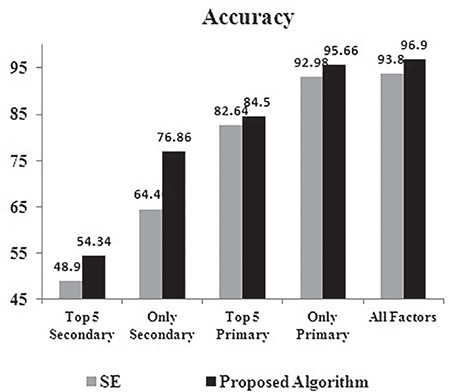
Accuracy of the classifiers – stacked ensemble vs proposed algorithm

The analysis of accuracy for SE and the proposed algorithm is depicted in Figure 5. Among the five experiments conducted, the accuracy was at the minimum for the experiment conducted with only the top five secondary risk factors. There was a big jump in accuracy when other secondary risk factors were also used for training the algorithms. The improvement in accuracy of the proposed algorithm reached the maximum of 12% when only the secondary risk factors were used for training. This implies that, when only trivial features are available, the proposed algorithm can still perform much better than SE. This is mainly due to the reason that the proposed algorithm is not affected much by overfitting or underfitting. When all the factors are used for training, there is an improvement of more than 3% in accuracy over SE. When only the primary risk factors were used for training, the accuracy of the proposed algorithm was just 1.3% below the accuracy of the proposed algorithm when all the risk factors were used. Hence the contribution of the secondary risk factors in PTB is not significant. Even the maximum accuracy of 93.8% achieved by SE when all the factors are used for training is 1.8% less than the accuracy achieved by the proposed algorithm with only primary factors. Hence, for high-dimension datasets also, the proposed algorithm can use minimal features and achieve better accuracy than SE.

**Figure 6:**
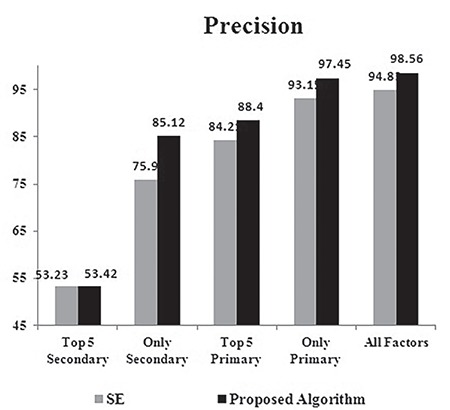
Precision of the classifiers – stacked ensemble vs proposed algorithm

The analysis of precision for SE and the proposed algorithm is depicted in Figure 6. The observed values of precision are also in similar lines of accuracy. The precision of the proposed algorithm reached the peak value of 98.56% when all the risk factors were used for training. The high value of precision for the proposed algorithm implies that the number of false positives was less. The high precision implies that most of the TB cases were predicted as NBs. This avoids unnecessary treatment being given to expectant mothers who would otherwise have undergone treatment. The improvement in precision reached a maximum of 10% when only secondary risk factors were used for training. Even with trivial factors, the proposed algorithm performed better than SE.

**Figure 7:**
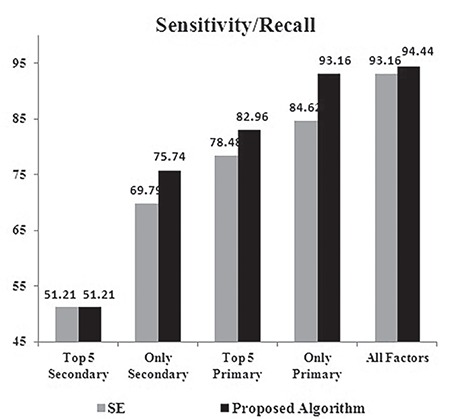
Sensitivity of the classifiers – stacked ensemble vs proposed algorithm

The analysis of sensitivity for SE and the proposed algorithm is depicted in Figure 7. The high value of sensitivity for the proposed algorithm implies that the number of false negatives was less. The high sensitivity implies that most of the PTB cases were predicted as PTBs. This indicates that the patients who need immediate medication are not ill-affected by the predictions of the proposed algorithm. The improvement in sensitivity reached the maximum of 8.5% when only primary risk factors were used for training. When the top five secondary risk factors were used for training, there was no improvement in sensitivity.

**Figure 8:**
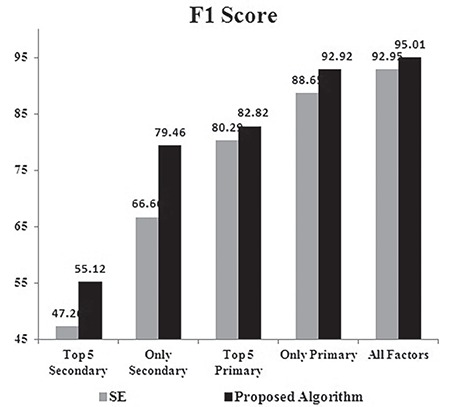
F1 score of the classifiers – stacked ensemble vs proposed algorithm

The analysis of F1 scores for SE and the proposed algorithm is depicted in Figure 8. The F1 score is the harmonic mean of precision and sensitivity. As the proposed algorithm achieved improvement in both precision and sensitivity, its F1 score was also better than that of SE for all five experiments. The F1 score reached the minimum when only the top 5 secondary risk factors were used to train the algorithms. The difference in the F1 score of SE and the proposed algorithm was as high as 13% when only the secondary risk factors were used for training. The F1 score reached the maximum when all the risk factors were used for training.

**Figure 9:**
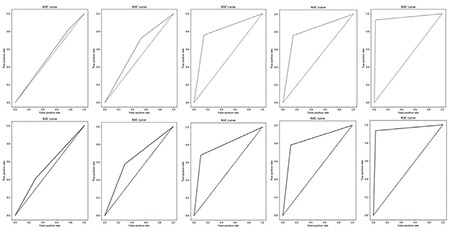
ROC curves for stacked ensemble (first row) and the proposed algorithm (second row)

ROC curves were drawn to analyze the AUC. The ROC for SE and the proposed algorithm for the five experiments are depicted in Figure 9. The set of graphs in the first row correspond to SE and the set of graphs in the second row correspond to the proposed algorithm. In the below graphs, the middle way mark of 50% is represented as dotted lines. As expected, the AUC reached the minimum when only the top five secondary risk factors were used to train the algorithms. The AUC increased with the number of critical factors used for training. The greater the number of critical factors used for training, the greater is the AUC. It reached a peak for both SE and the proposed algorithm when all risk factors were used for training. The minimum values of AUC for the top 5 secondary risk factors imply that the true positive rate did not reach the peak even if the false positive rate reached the minimum. This means that false negatives were high in the prediction. From the perspective of PTB, this is alarming. High values of false negatives imply that a patient who needs immediate attention and treatment may not receive treatment.

The application of the innovative SE algorithm for predicting PTB achieved better performance than SE for all the experiments conducted in this study. For all the performance metrics considered in this study, the innovative SE algorithm is way ahead when compared with the traditional SE algorithm. Primary risk factors play a major role in predicting PTB. When secondary factors were used along with primary risk factors, the performance metrics improved marginally (little more than 1%). Hence, using only primary risk factors with the proposed algorithm is the efficient method for PTB prediction. The time complexity of the proposed algorithm with different sets of factors can be considered for future work. The accuracy can be further improved by using a large number of base learners and combination schemes because the proposed algorithm is scalable in these terms. Finding the optimal number of base learners and combination schemes is also an interesting area to explore further. In order to increase the clinical use of this algorithm, we are considering the possibility of designing a mobile app with a wrapper around the algorithm. The mobile app allows physicians to enter the results of clinical tests of expectant mothers using an interface and provides the corresponding prediction. This mobile app hides the complexities of the statistical methods from the end user and thus greater numbers of physicians can benefit from this algorithm. We are also exploring if this algorithm can be enhanced and extended to analyze other maternal complications.

## Figures and Tables

**Table 1:**
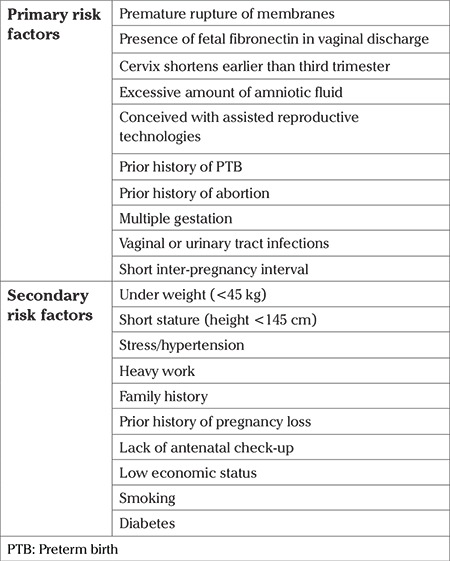
Risk factors associated with PTB (13,14)

**Table 2:**
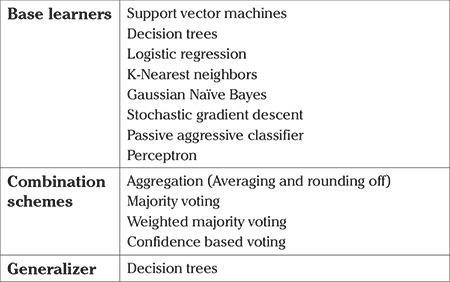
Classifiers used in different tiers

**Table 3:**
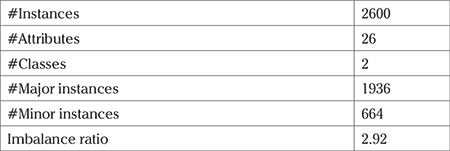
Description of the dataset

**Table 4:**
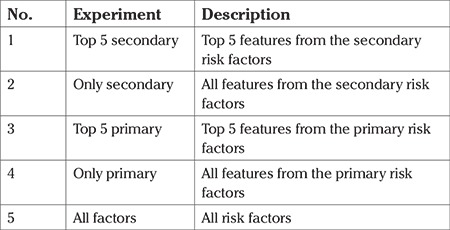
List of experiments

**Table 5:**
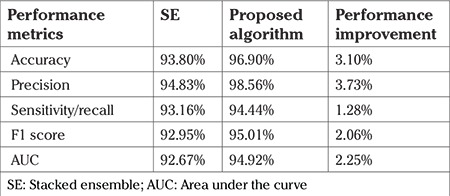
Summary of the results
